# Extraordinary Transport Characteristics and Multivalue Logic Functions in a Silicon-Based Negative-Differential Transconductance Device

**DOI:** 10.1038/s41598-017-11393-9

**Published:** 2017-09-11

**Authors:** Sejoon Lee, Youngmin Lee, Changmin Kim

**Affiliations:** 1 Department of Semiconductor Science, Dongguk University - Seoul, Seoul, 04623 Korea; 2 Quantum-Functional Semiconductor Research Center, Dongguk University - Seoul, Seoul, 04623 Korea

## Abstract

High-performance negative-differential transconductance (NDT) devices are fabricated in the form of a gated p^+^-i-n^+^ Si ultra-thin body transistor. The devices clearly display a Λ-shape transfer characteristic (*i.e*., Λ-NDT peak) at room temperature, and the NDT behavior is fully based on the gate-modulation of the electrostatic junction characteristics along source-channel-drain. The largest peak-to-valley current ratio of the Λ-NDT peak is greater than 10^4^, the smallest full-width at half-maximum is smaller than 170 mV, and the best swing-slope at the Λ-NDT peak region is ~70 mV/dec. The position and the current level of the Λ-NDT peaks are systematically-controllable when modulating the junction characteristics by controlling only bias voltages at gate and/or drain. These unique features allow us to demonstrate the multivalue logic functions such as a tri-value logic and a quattro-value logic. The results suggest that the present type of the Si Λ-NDT device could be prospective for next-generation arithmetic circuits.

## Introduction

For the last two decades, several types of novel-functional electronic devices have been proposed and demonstrated on a variety of device architectures so as to huddle up the limitation of conventional complementary metal-oxide-semiconductor (CMOS) devices^1–4^. For example, one of the most promising scheme is the negative-differential transconductance (NDT) and the negative-differential resistance (NDR) devices, in which quantum mechanical characteristics (*e.g*., resonant tunneling^5–9^, single-electron tunneling^10–18^, band-to-band tunneling^19–21^ *etc*.) and/or ambipolar carrier actions^22–25^ are implemented. In the operation point of view, the NDT/NDR devices exhibit the extraordinary transfer- and/or output-characteristics. Namely, the devices show a current or a voltage oscillation peak at the specific bias point. This enables us to demonstrate some of astonishing functionalities beyond the binary logic system. For instance, multiple logic functions^26–28^, multivalued logics^29–31^, and stochastic data processes^32^ are prominent representatives that can put a step closer to the future electronic computing system. Furthermore, since the usage of the NDT/NDR device allows a high-speed operation of the electronic circuit system (*e.g*., high-frequency oscillators^33–35^, high-speed multiplexers^36, 37^, and fast logic switches^38, 39^ *etc*.), exploiting the high-performance NDT/NDR devices could be of major importance in the next-generation ultra-large-scale integration technology. To realize highly-functional NDT/NDR devices, many of emerging materials (*e.g*., carbon nanotube^40^, graphene^8–11^, molybdenum disulfide^7, 12^, single molecule^41^ *etc*.) and semiconductor nanostructures have been employed in such a prospective concept of the device scheme. Regardless of the extensive efforts made to replace Si, however, technical and scientific knowledge accumulated on Si still can offer an advantage for rapid innovations^42, 43^. These backgrounds prompt a systematic study on highly-functional Si NDT/NDR devices that are not only compatible to CMOS technology but also reliable for high reproducibility.

In light of this, we have fabricated and characterized the Si NDT transistors that can be utilized for next-generation multivalue arithmetic circuits. In this article, we report data on the extraordinary characteristics of the high performance Si NDT transistors, which were fabricated using a CMOS-compatible device fabrication process. The electrical transport properties and the multivalue logic functions are thoroughly examined, and the transport mechanisms are discussed in detail.

## Experimental Details

**Figure 1:**
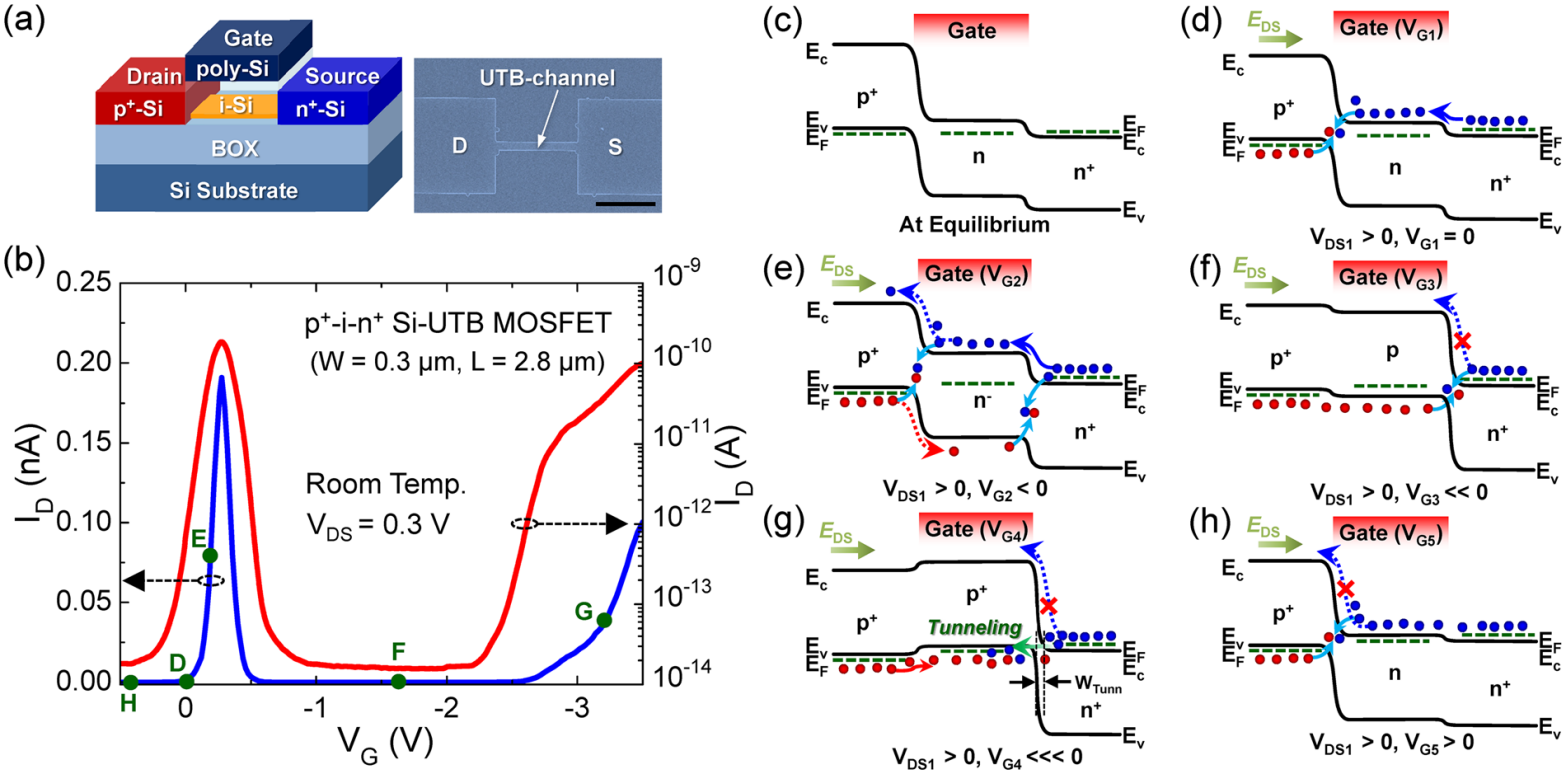
(**a**) Schematic of the gated p^+^-i-n^+^ Si UTB transistor (left-hand-side panel) and Scanning electron microcopy image of the patterned Si UTB channel (scale bar: 2 μm) (right-hand-side panel), (**b**) NDT characteristics in I_D_-V_G_ curves of the gated p^+^-i-n^+^ Si UTB transistor, (**c**–**h**) Energy band diagrams at various bias conditions, representing a possible transport mechanism of the NDT characteristics in the gated p^+^-i-n^+^ Si UTB transistor: (**c**) thermal equilibrium, (**d**) V_D1_ > 0 and V_G1_ = 0 V, (**e**) V_D1_ > 0 and V_G2_ < 0 V, (**f**) V_D1_ > 0 and V_G3_ ≪0 V, (**g**) V_D1_ > 0 and V_G4_ ≪<0 V, and (**h**) V_D1_ > 0 and V_G5_ > 0 V. E_c_, E_v_, and E_F_ labeled in each band diagram denote the conduction band minimum, the valence band maximum, and the Fermi level, respectively.

The NDT devices were fabricated in the form of the gated Si p^+^-i-n^+^ ultra-thin body (UTB) metal–oxide–semiconductor field-effect transistor (MOSFET) on a silicon-on-insulator (SOI) substrate (*t*_BOX_ ≈ 300 nm) (left-hand-side panel of Fig. 1(a)). To construct such a device structure, we used the undoped (100) Si layer (*n*_hole_ ~ 5 × 10^15^ cm^−3^) of the SOI substrate as a starting material. For convenience, we refer the undoped Si layer as i-Si. For the formation of the UTB channel, first, the i-Si layer was thinned down to ~20 nm by successive thermal oxidation and chemical deoxidation. Next, the channel areas (W: 0.3–2.0 μm, L: 2.8 μm) were patterned by using conventional lithography techniques (see the right-hand-side panel of Fig. 1(a)). For further thinning of the SOI thickness (<10 nm), thereafter, we carried out local oxidation of silicon at the channel regions. During this step, ~5-nm-thick gate oxide was created; hence, the thickness of the UTB channel became less than 5 nm while that of the source/drain remained thick enough to minimize parasitic resistances. To prevent the gate leakage, we subsequently deposited an additional silicon dioxide layer (*t*_ox_ ≈ 20 nm) through the low-pressure chemical vapor deposition method. Then, the p^+^-type drain (*p* ~ 10^20^ cm^−3^) and the n^+^-type source (*n* ~ 10^20^ cm^−3^) were formed by ion implantation of BF_2_^+^ and P^+^, respectively. Finally, the n-type polycrystalline Si gate and the Al electrode were constructed *via* conventional MOSFET fabrication processes. The electrical properties of the Si p^+^-i-n^+^ UTB-channel MOSFETs were measured at room temperature by using a Keysight B1500A device parameter analyzer and an Agilent DSO-6104A oscilloscope system.

## Results and Discussion

Figure 1(b) shows the drain current *vs*. gate voltage (I_D_–V_G_) characteristic curves at room temperature of the fabricated Si p^+^-i-n^+^ UTB-channel MOSFET. Under the drain-source voltage (V_DS_) of 0.3 V, the device clearly exhibits an N-shape transfer characteristic (*i.e*., NDT effect) with a Λ-shape peak at V_G_ = 0–|−0.5| V. For convenience, we refer this peak as a Λ-NDT peak in the present study. The full-width at half-maximum (FWHM) of the Λ-NDT peak is less than 170 mV, and the peak-to-valley current ratio (PVCR) is greater than 10^4^. Such a sharp and prominent Λ-NDT feature can be of great benefit for the high-speed analog circuits^33–35^ and the novel functional digital circuits^26–32^. As a primary task, thus, understanding the physical mechanism of the clear NDT effect is essential for more feasibility and reproducibility.

We, therefore, firstly explain the transport mechanism of the device to help understand operation schemes of our NDT transistor. Figure 1(c–h) illustrate the carrier transport behaviors of the gated p^+^-i-n^+^ Si-UTB transistor under various bias conditions. At thermal equilibrium, a large built-in potential would be formed at the junction between the drain and the channel because the lightly-doped p^–^channel becomes an n-type due to the band-bending effect from the work-function difference between the n-type polycrystalline-Si gate (Φ_gate_ ~ 4.0 eV) and the p^−^- channel (Φ_ch_ ~ 4.94 eV for p^−^Si). (Fig. 1(c)). In addition, a small hump would be formed at the junction between the channel and the source because of the difference in electron concentrations at the n-channel and the n^+^-source. The potential barrier at each side will be slightly lowered when the forward bias voltage is applied to the drain-source (*i.e*., V_DS1_ > 0) (Fig. 1(d)). At this bias point, despite no gate bias (*i.e*., V_G1_ = 0), a small current can flow through the channel because of carrier recombination and weak diffusion at both p^+^-n and n-n^+^ junctions, respectively (*e.g*., Point D in Fig. 1(a)).

Here, one can easily create the NDT feature by changing the magnitude of |V_G_| because the gate driving force is very strong in the UTB-based MOS stack (*i.e*., explicit control of the accumulation-depletion-inversion modes by |V_G_| in the UTB-channel MOSFETs)^44, 45^. For example, when applying a negative gate voltage (*i.e*., V_G2_ < 0), I_D_ will start to increase because −|V_G2_| reduces the electron concentration at the channel and eventually gives rise to the increase in diffusion/drift currents through the source-channel-drain (Fig. 1(e)) (*e*.*g*., Point E in Fig. 1(a)). When the magnitude of |−V_G_| further increased (*i.e*., V_G3_  ≪ 0), however, I_D_ will drastically decrease because −|V_G3_| accumulates plenty of holes in the channel. Namely, |V_G3_| will increase the potential barrier height at the channel-source (*i*.*e*., p-n^+^) junction as large as the diffusion/drift action could be inhibited (Fig. 1(f)). As a result, the reverse saturation would occur at the channel-source junction; hence, I_D_ will rapidly decrease at V_G3_ (*e.g*., Point F in Fig. 1(a)). Such a sudden drop of I_D_ causes a Λ-NDT phenomenon in the present type of the NDT transistor.

Through keeping on increasing −|V_G_| (*i.e*., V_G4_ ⋘ 0), the electrons can transfer from the source to the channel *via* band-to-band tunneling (BTBT). In other words, the BTBT event will occur under −|V_G4_| because large −|V_G_| populates the channel with abundant hole carriers as much as the depletion width becomes thin enough to allow BTBT at the p^+^-n^+^ junction (Fig. 1(g)). At this bias stage, I_D_ will significantly increase due to both the hole drift at p^+^-p^+^ and the electron tunneling event at p^+^-n^+^ (*e.g*., Point G in Fig. 1(a)). All of the above allow the gated Si p^+^-i(p^−^)-n^+^ UTB-channel MOSFET to exhibit the N-shape transfer characteristic in the negative V_G_ region. At the positive V_G_ region (*i*.*e*., V_G5_ > 0), the value of I_D_ would remain low (*e.g*., Point H in Fig. 1(a)) because +V_G_-induced electrons in the channel increases potential barriers at the drain-channel (*i.e*., p^+^-n) junction (Fig. 1(h)).

Here, we point out the statistical uncertainty of BTBT at the higher |V_G_| region (*e.g*., at |V_G_| ≫ |−2| V in Fig. 1(a)). To perform BTBT, in fact, four necessary and sufficient conditions must be simultaneously satisfied: (i) the occupied energy states should exist in the reservoir to supply charge carriers, (ii) the unoccupied states also should exist in the charge collection region, (iii) the tunnel barrier width should be thin enough to ensure a finite tunneling probability, (iv), the momentum must be conserved during tunneling events. When fabricating the integrated circuit, however, the BTBT probability in semiconductor junction devices would be different from each other because the above conditions are very sensitive to both the energy perturbation and the thermal fluctuation. As a result, the tunneling current would be nonidentical for every device, leading to a vague output in the integrated circuit.

On the other hand, the NDT effect at the Λ-shaped peak region (*e.g*., at V_G_ = 0–|−0.5| V in Fig. 1(a)) is reliable and reproducible for every device because the behavior occurs on the basis of only gate-controlled ambipolar carrier actions at the junction areas (*i.e*., gate control of ‘recombination → diffusion/drift → reverse saturation’), as discussed earlier. Furthermore, fast switching of the positive-to-negative differential transconductance at the Λ-shaped peak region is beneficial for future high-speed and functional circuit applications. Therefore, from now on, we emphasize the features of the Λ-NDT peaks, which can be effectively demonstrated and modulated by junction dynamics in the device.

**Figure 2:**
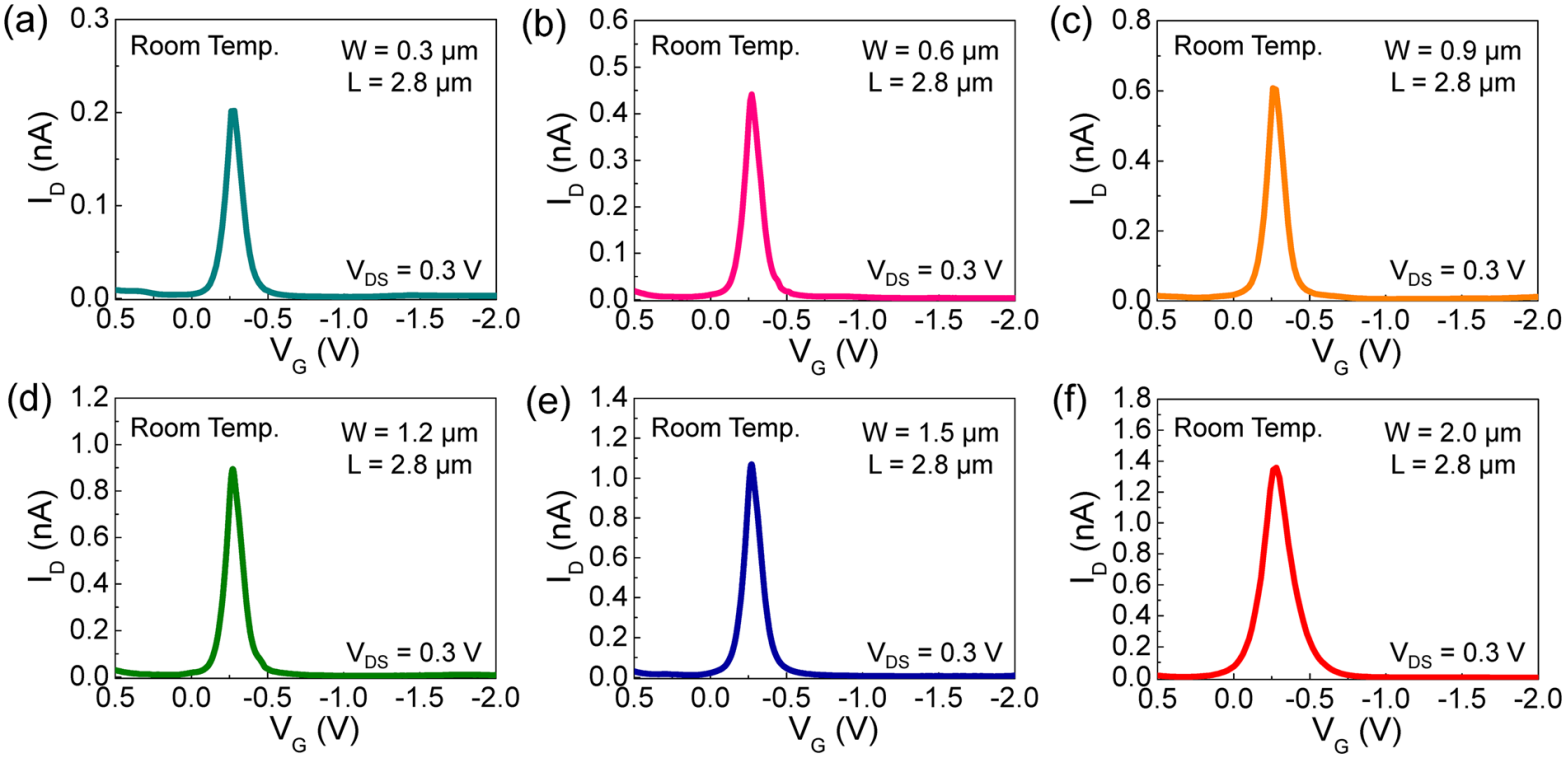
Λ-NDT characteristics in I_D_-V_G_ curves of the gated p^+^-i-n^+^ Si UTB transistors with different channel sizes: (**a**) W: 0.3 μm, L: 2.8 μm, (**b**) W: 0.6 μm, L: 2.8 μm, (**c**) W: 0.9 μm, L: 2.8 μm, (**d**) W: 1.2 μm, L: 2.8 μm, (**e**) W: 1.5 μm, L: 2.8 μm, and (**f**) W: 2.0 μm, L: 2.8 μm.

From the Si p^+^-i-n^+^ gated-transistors fabricated through the aforementioned procedures, more than 65% of the devices showed clear NDT characteristics at room temperature. As shown in Fig. 2(a–f), the devices clearly exhibit the Λ-NDT peak in their transfer characteristic curves. Regardless of the channel size (*i.e*., W/L), the Λ-NDT peak clearly appears at V_G_ = |0–−0.5| V, while the peak current increases with increasing channel width. This verifies our NDT transistors to hold promise for future CMOS-compatible novel functional circuit applications. The magnitude of PVCR is no less than 10^4^ for all devices, and the value of FWHM is~175 mV on average.

**Figure 3:**
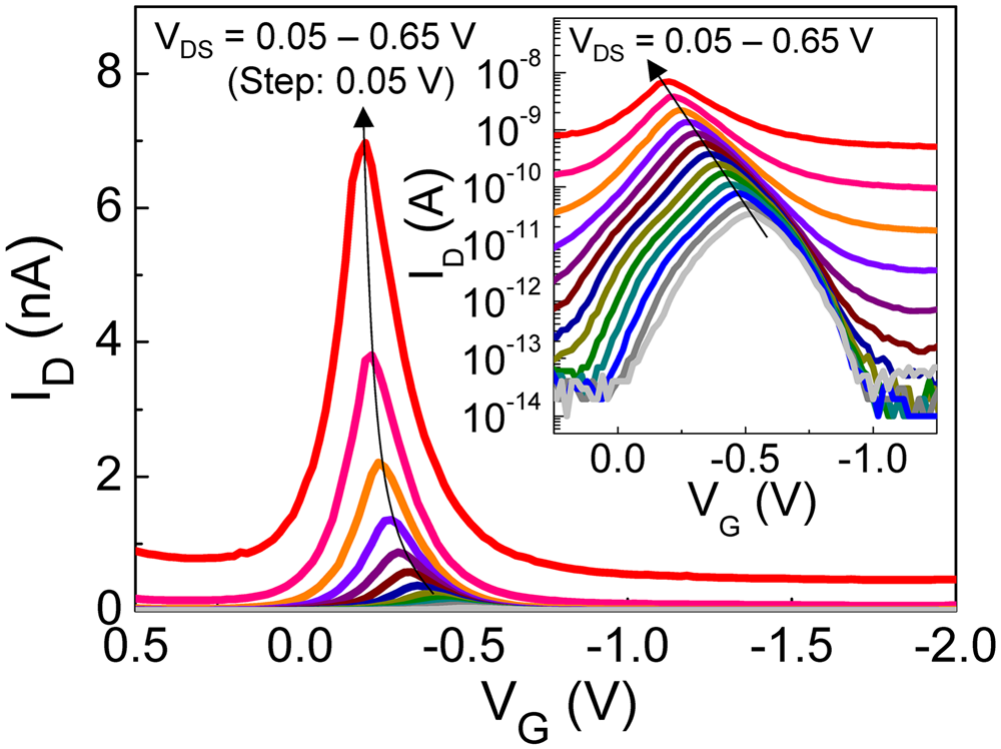
Dependence of the Λ-NDT characteristics on V_DS_ ranging from 0.05 to 0.65 V. The inset displays a semi-logarithmic plot of I_D_-V_G_ curves at V_DS_ = 0.05–0.65 V.

Since the junction-depletion characteristics depend on both the Fermi potential inside the channel and the built-in potential at the channel edge, one may expect that the Λ-NDT conditions (*i.e*., recombination → diffusion/drift → reverse saturation) can be modulated by controlling either of V_G_ or V_DS._ We, accordingly, measured the I_D_-V_G_ characteristics at various V_DS_ to investigate the effect of bias conditions on the modulation of Λ-NDT peaks (Fig. 3). As the magnitude of +V_DS_ increases, the peak current at the Λ-NDT region is exponentially increased because the large +V_DS_ enhances the drift action at the source-channel-drain junction. In addition, the Λ-NDT peak position systematically shifts toward the lower |−V_G_| region with increasing +V_DS_ (see also the inset of Fig. 3).

The precise control of NDT peaks in our Si p^+^-i-n^+^ UTB MOSFET is quite similar to that in highly-functional single electron/hole transistors that were devised with ultra-small quantum dots (*e.g*., d_dot_ < 5 nm)^13–18^. In this otherwise quantum nature-free NDT device (*e.g*., no quantum dot *etc*.), however, we explicitly demonstrated the systematic modulation of the Λ-NDT peak through only controlling the electrostatic junction characteristics. Namely, the position and the magnitude of the Λ-NDT peak can be precisely controlled through modifying the potential profile for the NDT condition^25^. For instance, when a lower |+V_DS_| is applied to the device, a larger |−V_G_| is necessary to accumulate plenty of holes in the channel for performing the Λ-NDT phenomenon (*i.e*., switching of ‘diffusion/drift → reverse saturation’ by |−V_G_|), and *vice versa* at a higher |+V_DS_|.

**Figure 4:**
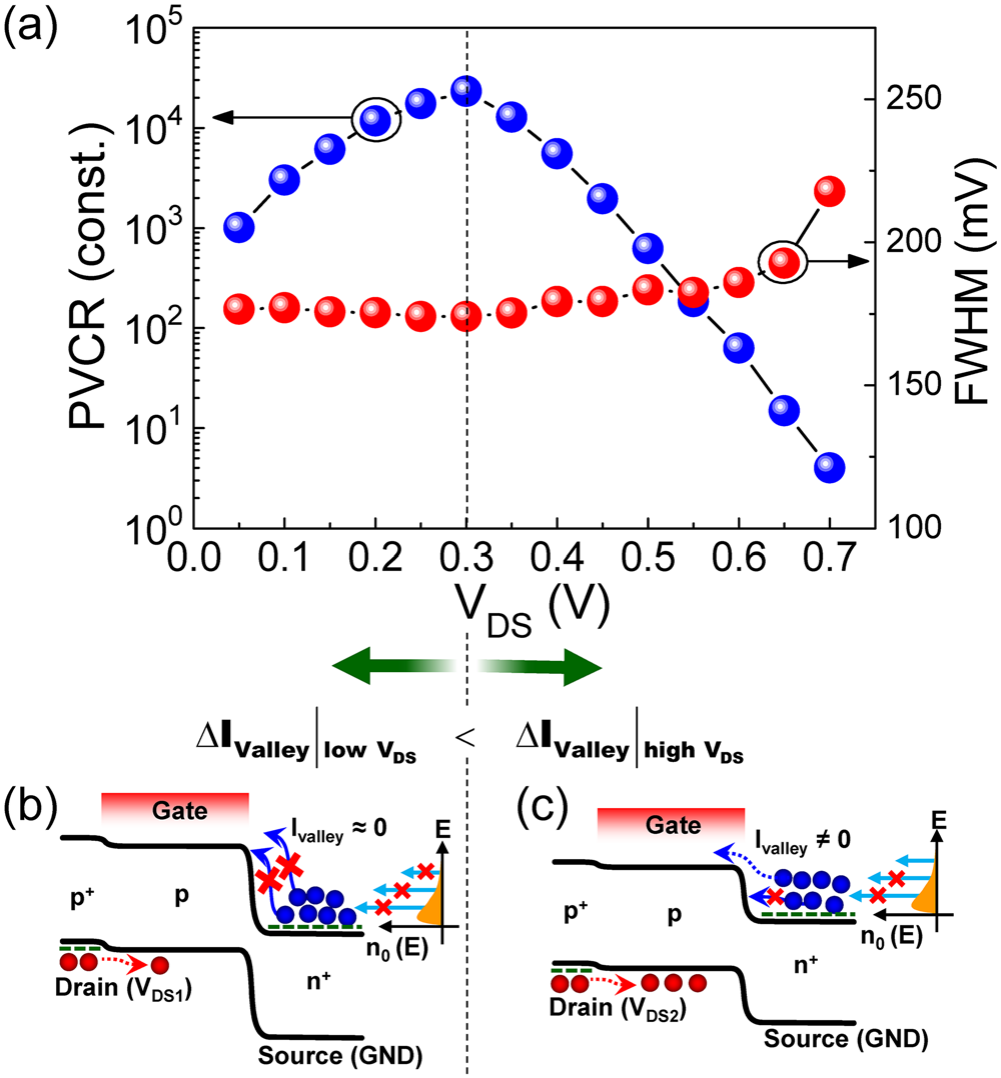
(**a**) PVCR and FWHM of the Λ-NDT peaks as a function of V_DS_. (**b**–**c**) Potential profiles along the drain-channel-source region at different V_DS_ bias conditions: (**b**) Lower V_DS_ (*e.g*., V_DS1_ ≤ 0.3 V) and (**c**) Higher V_DS_ (*e.g*., V_DS2_ ≥ 0.35 V). The rightmost graph of ‘n_0_ (E) *vs*. E’ represents the electron distribution function of n^+^-Si at room temperature.

When using the NDT device for the electronic circuits, the values of PVCR and FWHM play key factors because those are closely related to both the on/off ratio and the switching speed of the device. Thus, we assess the dependences of PVCR and FWHM on the bias conditions. As can be seen from Fig. 4(a), the bias voltage of V_DS_ strongly affects the value of PVCR. With increasing V_DS_ up to 0.3 V, the magnitude of PVCR increases and reaches ~2 × 10^4^, whereas that monotonically decreases when V_DS_ exceeds 0.35 V. This can be explained by the variation of the off-current upon varying V_DS_. When V_DS_ is low (*e.g*., V_DS_ ≪ 0.3 V), the built-in potential at the channel-source junction (V_bi(c-s)_) is still high enough to cut off the carrier transport thorough the channel (*i.e*., off-current = very low) (Fig. 4(b)). In this case, since the on-current increases with increasing V_DS_ (*e*.*g*., up to 0.3 V), the magnitude of PVCR becomes higher. When V_DS_ is high (*e.g*., V_DS_ > 0.3 V), however, the barrier height of V_bi(c-s)_ is decreased as low as a few of electrons can flow from the source to the channel (*i.e*., off-current ≠ low) (Fig. 4(c)). In this case, the value of off-current becomes higher and higher with increasing V_DS_; hence, the magnitude of PVCR decreases in spite of the increase in on-current at higher V_DS_. Different from the behavior of PVCR, the effect of V_DS_ is insignificant on the magnitude of FWHM (Fig. 4(a)) because the capacitive coupling strength of the UTB gate stack is much stronger than that of the drain-channel-source junction.

**Figure 5:**
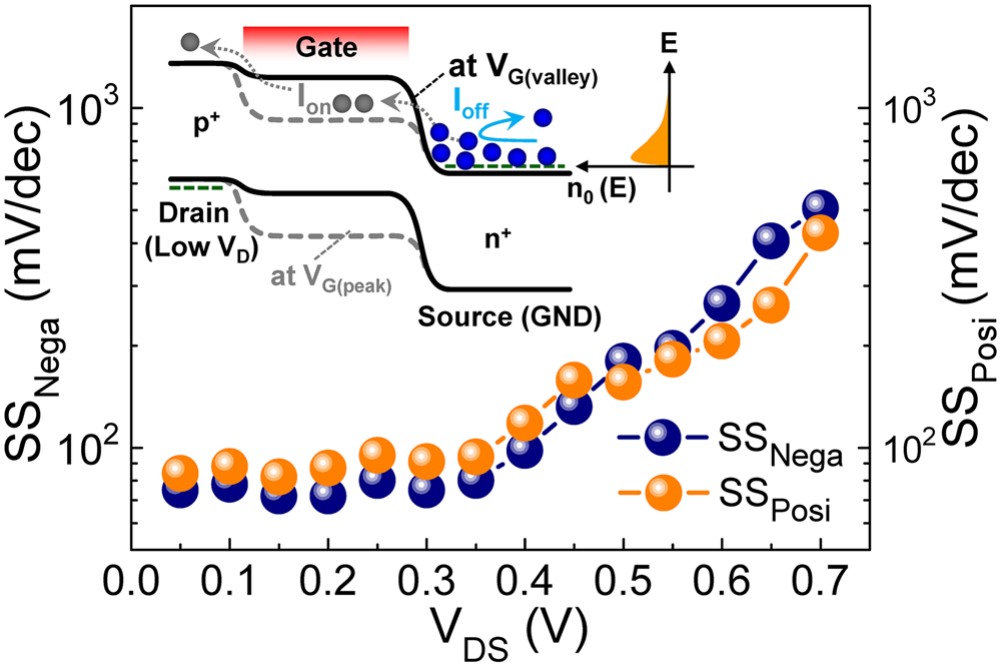
SS_Nega_ and SS_Posi_ of the Λ-NDT peaks as a function of V_DS_. The inset illustrate the energy band diagram that represents the V_G_-controlled fast switching behavior of the device (High |V_G_|: solid lines, Low |V_G_|: dashed lines).

Another important factor of the NDT device is its V_G_-tunable swing-slope (SS) at the NDT peak region because SS is a key parameter of the device performance to produce a high speed on/off operation upon the input signals. The dependences of SS values on V_DS_ are shown in Fig. 5. The swing slopes at both the positive- and the negative-differential conductance regions (*i.e*., SS_Nega_ and SS_Posi_) show a similar behavior because those are mostly influenced by strong gate-tuning of V_bi(c-s)_ (*i.e*., fast switching of on/off operations by V_G_ in the UTB gate stack) (see the inset of Fig. 5). The best value of SS is ~70 mV/dec at V_DS_ < 0.35 V, which is comparable to that in the state-of-the-art Si MOSFETs^46–50^. When V_DS_ exceeds 0.4 V, however, the value of SS begins to increase because of the increased off-current at higher V_DS_, as discussed above.

**Figure 6:**
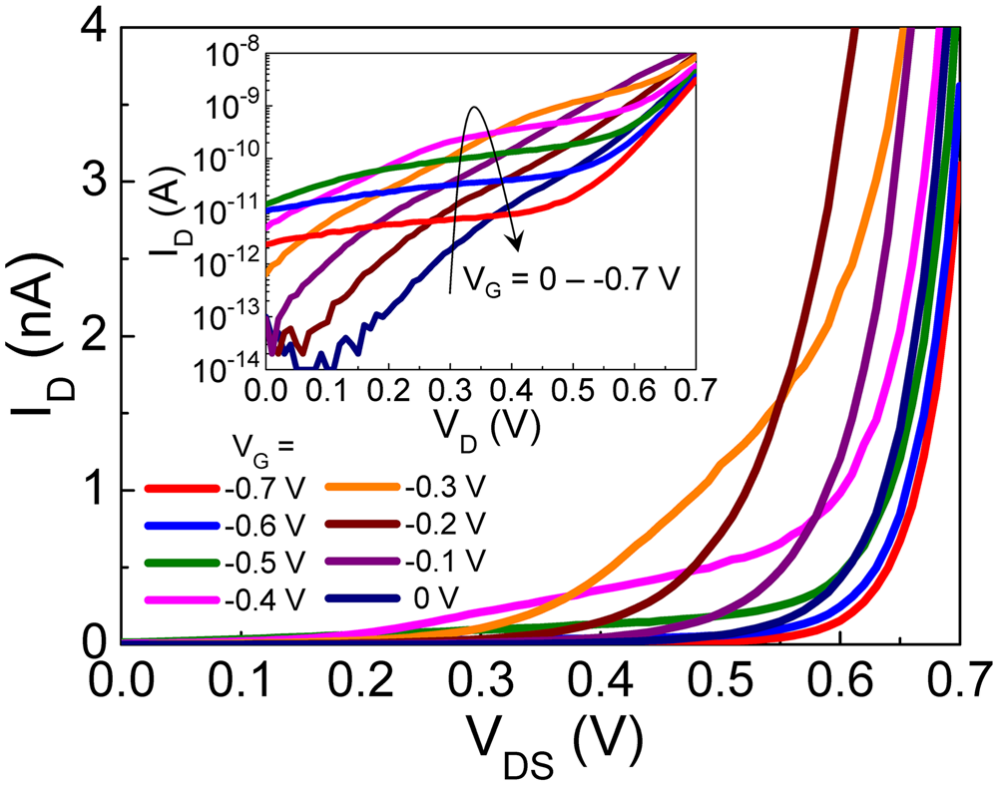
I_D_-V_DS_ characteristic curves at various V_G_ near the Λ-NDT peak region (*i*.*e*., V_G_ = 0 to −0.7 V). The inset displays a semi-logarithmic plot of I_D_-V_DS_ curves at V_G_ = 0–−0.7 V.

Figure 6 shows the I_D_-V_D_ characteristic curves of the device at various V_G_ near the Λ-NDT peak region. At V_G_ = 0 V, the device exhibits a typical diode-like feature because the p^+^-i-n^+^ junction is formed along the drain-channel-source region. As the magnitude of |−V_G_| increases up to |−0.4 V|, the turn-on voltage decreases and the on-state current increases because -V_G_ would induce hole accumulation in the channel and could reduce total V_bi_ along drain-channel-source (*i*.*e*., p^+^-p-n^+^). When |−V_G_| is further increased (>|−0.5| V), however, the turn-on voltage rapidly increases because the large magnitude of |−V_G_| would accumulate more holes inside the channel area; hence, total V_bi_ would increase particularly at the junction between channel and source (*i*.*e*., p^++^-n^+^). In addition, the device displays the current staircases (CSs) at V_G_ = −0.5–−0.7 V (see the inset of Fig. 6) due to the suppression of carrier conduction at the NDT region. As |V_G_| increases, the range of CS becomes wide, and the current level of the plateau goes down. Namely, the knee position of CS shifts stepwise toward the lower V_D_ and the lower I_D_.

**Figure 7:**
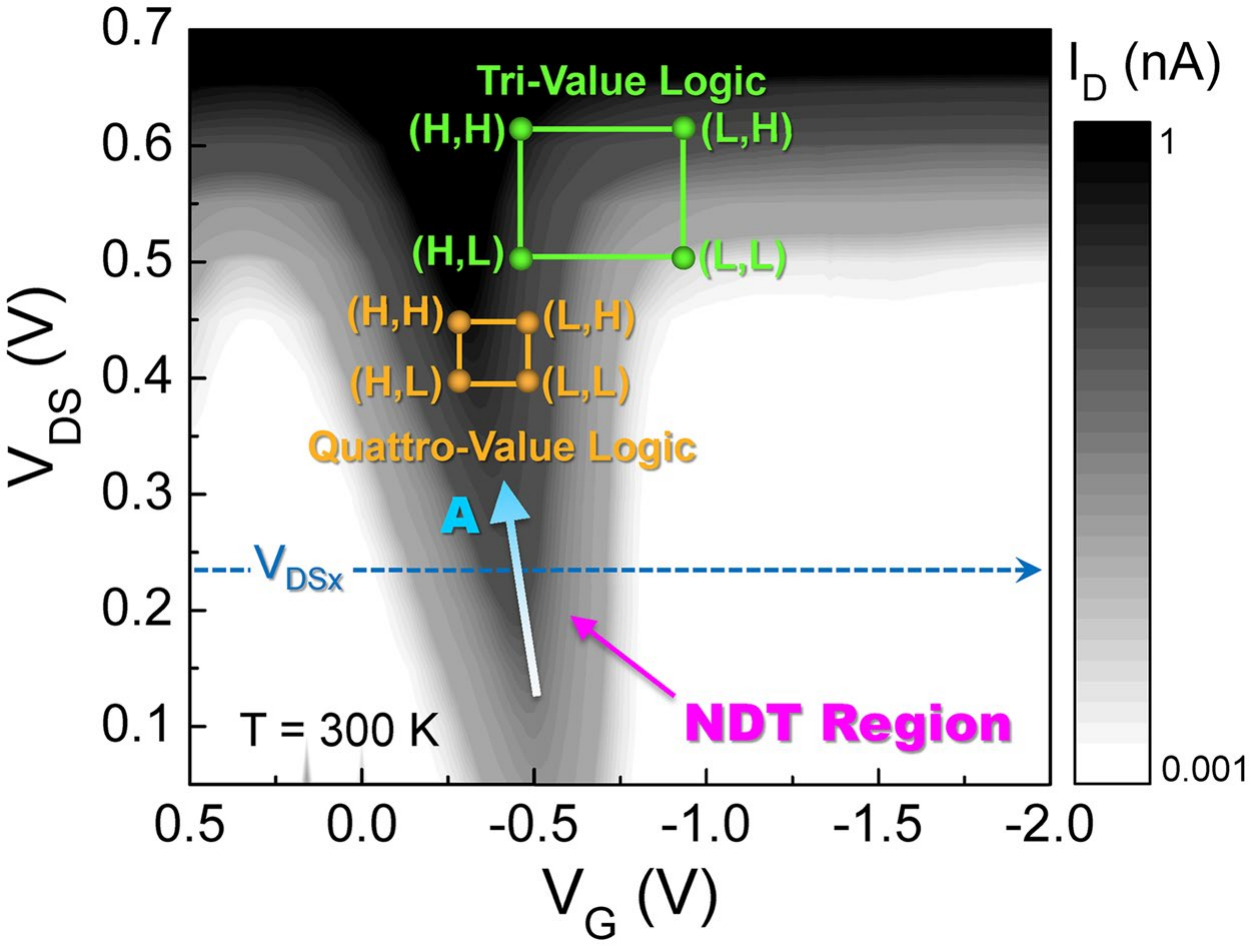
Contour plot of I_D_ as functions of V_G_ and V_DS_ for the gated p^+^-i-n^+^ Si UTB transistor. The green and orange dots pointed in the contour plot depict the bias points for performing the tri- and quattro-value logic functions using a unit device of our Λ-NDT transistor, respectively.

The stepwise-shifts of both NDT peaks and CS plateaus are useful for the circuit application of the NDT device because it can provide multiple operation points for logical functions at a wide range of voltages^28^. Such remarkable tunabilities of NDT and CS can be traced at a glance by measuring the charge diagram of the device. As can be seen from the contour plot of I_D_ as functions of both V_G_ and V_DS_ (Fig. 7), both the Λ-NDT and the CS characteristics are systematically modulated by V_G_ and V_D_. For example, at the fixed V_DS_ (*e.g*., V_DSx_), the color of I_D_ is changed along −|V_G_| direction (*i*.*e*., white → gray → black → gray → white). This corresponds to the −|V_G_|−dependent change in the current level of I_D_, indicating the appearance of the Λ-shaped I_D_ peak (*i*.*e*., Λ-NDT). As the magnitude of V_DS_ increases, the Λ-NDT region is extended toward the A direction. The extended Λ-NDT region is fairly long and inversely cuspidal, where the stepwise shifts of the Λ-NDT peaks and the CS plateaus occur, as confirmed in Figs 3 and 6.

**Figure 8:**
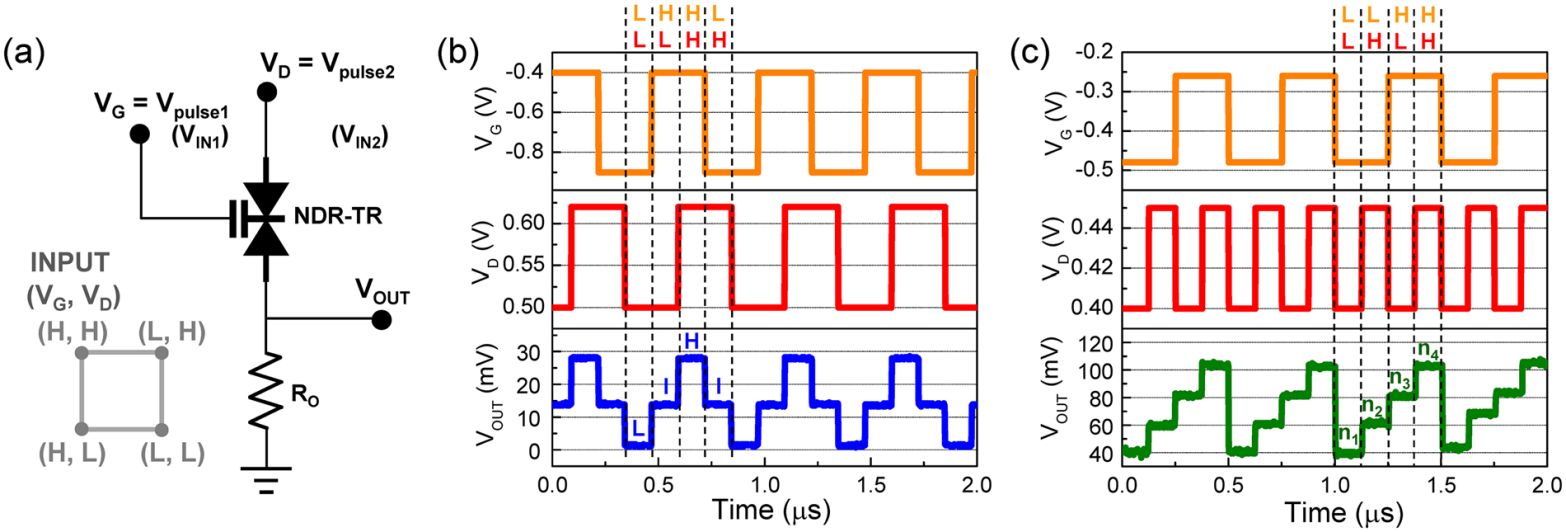
(**a**) Definition of the bias points (gray color) and the circuit configuration of the one-transistor multivalue-logic gate using an NDT device. To convert the output current to the voltage output, the output resistance of ~10 MΩ is used by connecting an additional MOSFET as an active load. (**b**) Transient waveforms for the tri-value logic function. (**c**) Transient waveforms for the quattro-value logic function.

Thanks to the appearance of the extended Λ-NDT peak region, one can choose many of the operation points from a unit device for the demonstration of multivalue logic functions. For example, when using our NDT device as a one-transistor logic gate, two input-bias voltages (*i.e*., V_IN1_ = V_G_ and V_IN2_ = V_D_) can be selected at specific bias points for demonstrating different multivalue logic functions (see also Fig. 8(a)). Following this way, as depicted in Fig. 7, a tri-value and a quattro-value logic functions can be chosen as possible examples of one-transistor multivalue logics. Figure 8(b) and (c) display the measured transient waveforms of the tri-value and the quattro-value logics, respectively. The voltage output (V_OUT_) clearly reveals a sequential count function of the multivalue upon varying V_G_ (=V_pulse1_) and V_D_ (=V_pulse2_). Although the output-voltage level is quite low because of the low current level at the NDT region, we believe that the multivalue logic functions can be effectively used for future highly-sensitive low-power arithmetic circuits.

Finally, we briefly state the speed limit of the NDT-based multivalue logic circuits. In our device, the channel conductance near the Λ-NDT peak is in the order of 10 s nS, which corresponds to the junction resistance (R_j_) of a few of hundreds MΩ. In addition, the junction capacitance (C_j_ ≈ 1/2·q/kT·τI_DQ_^51^, where k is the Boltzmann constant, T is the environmental temperature, I_DQ_ is the driving current at an operation point, and τ is the carrier lifetime) is determined to be ~2 fF, when assuming *τ* = 10^−7^ s^51^. Furthermore, since the gate capacitance (C_g_ = W∙L∙k_ox∙_ε_0_/t_ox_^51^, where k_ox_ is the relative dielectric constant of SiO_2_, ε_0_ is the vacuum permittivity, and t_ox_ is the thickness of SiO_2_) is ~1.2 fF for the present device (W = 0.3 μm, L = 2.8 μm, t_ox_ = 25 nm), the time constant (≈R_j_C_j_) of our Λ-NDT device can be estimated to be less than 0.1 μs. We can, therefore, deduce the intrinsic speed of the device to be no less than several tens of MHz. Although the intrinsic speed limit seems little low, the implementation of high-mobility device architectures (*e.g*., nanowire- or nanosheet-channel MOSFETs with a gate-all-around stack^52–55^) to the present type of the NDT device can be the next step to improve the speed of the NDT-based one-transistor multivalue logic circuits.

## Conclusion

The NDT devices were fabricated in the form of the Si p^+^-i-n^+^ UTB-channel MOSFETs. The devices clearly showed a Λ-shape NDT peak, at room temperature, with the extremely large PVCR (>10^4^) and the small FWHM (<170 mV). These features were universal for multiple devices that had been fabricated using an identical method (*i.e*., yield ~65%). The best value of SS at the Λ-shape NDT peak region was ~70 mV/dec. In addition, the Λ-NDT peaks were confirmed to be effectively modulated through the control of the junction characteristics by changing only V_G_ and/or V_DS_. Owing to the systematic modulation of the Λ-NDT peaks, we successfully demonstrated the multivalue logic functions (*e.g*., tri-value and quattro-value logics) on a single device as a one-transistor multivalue logic gate. These may offer potential applications for low power/high speed multivalue logics beyond the ordinary binary logic system.
